# Supplementary material for: A global analysis of extreme coastal water levels with implications for potential coastal overtopping
Source: Nat Commun. 2021 Jun 18;12:3775. doi: 10.1038/s41467-021-24008-9 (PMC8213734; doi:10.1038/s41467-021-24008-9)
Supplement: Supplementary file 1 — Supplementary Information [file 41467_2021_24008_MOESM1_ESM.pdf]

## Supplementary Material

# A global analysis of extreme coastal water levels with implications for potential coastal overtopping

Rafael Almar[1,*], Roshanka Ranasinghe[2,3,4], Erwin W.J. Bergsma[1], Harold Diaz[1], Angelique Melet[5], Fabrice Papa[1], Michalis Vousdoukas[6], Panagiotis Athanasiou[3,4], Olusegun Dada[7], Luis Pedro Almeida[8], and Elodie Kestenare[1]

[1]LEGOS (CNRS/IRD/CNES/Toulouse University), Toulouse, France

[2]Department of Coastal and Urban Risk @ Resilience, IHE Delft Institute for Water Education, P.O. Box 3015 2610 DA Delft, The Netherlands

[3]Harbour. Coastal and Offshore Engineering, Deltares, PO Box 177, 2600 MH Delft, The Netherlands

[4]Water Engineering and Management, Faculty of Engineering Technology, University of Twente, PO Box 217, 7500 AE Enschede, The Netherlands.

[5]Mercator-Ocean, Toulouse, France

[6] European Commission, Joint Research Centre (JRC), Ispra, Italy

[7]Federal University of Technology, Akure, Nigeria

[8]Universidade Federal do Rio Grande do Sul, Rio Grande, Brazil

Corresponding author [*]rafael.almar@ird.fr

**S1. Regional patterns of change in overtopping under global mean sea level rise**

Global mean sea level rise will increase overtopping occurrence. Increasingly, more regions will become vulnerable to overtopping. This change is not uniform globally and over the 21st century, and depends on the local coastal elevation. Overtopping at some low lying areas increases notably even for a small GMSLR (0.3 m above present level, corresponding to end of century projection under RCP 2.6), such as the Western Pacific islands and parts of northwestern Africa, while other regions, mainly in the Tropics, western USA, and far-eastern Russia, a moderate to high GMSLR (0.6 m and above present level, corresponding to end of century projection under RCP 4.5) is required for significant increases in overtopping,.

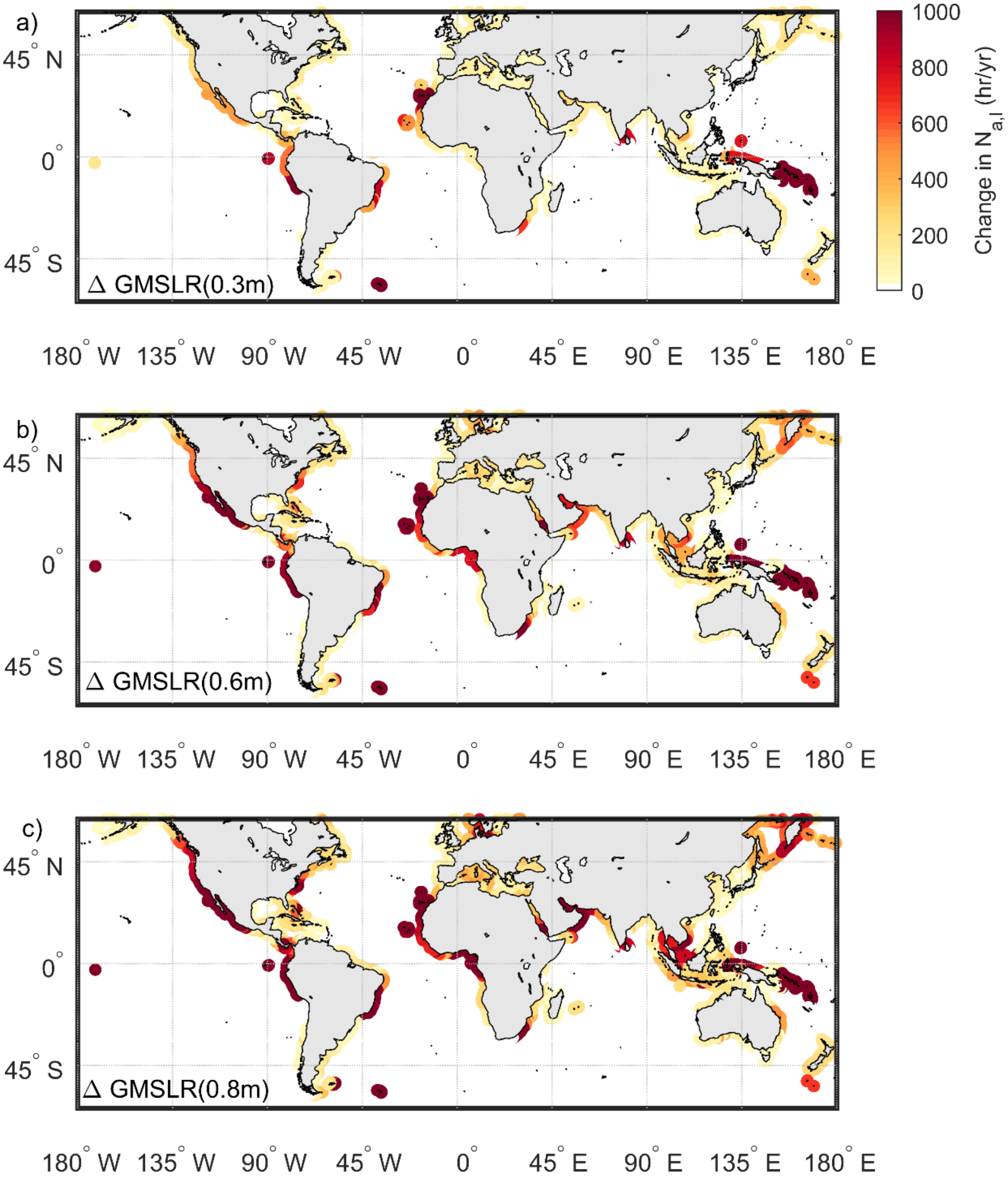


**Figure S1.** Regional differences in change (from present-day) of the annual number of overtopping hours ($N_{a,l}$) for global mean sea level rises of 0.3 m, 0.6 m and 0.8 m, corresponding respectively to end-century projections for RCP 2.6, RCP 4.5 and RCP 8.5 respectively.

**S2. Sensitivity of overtopping projections to different topography datasets**

The choice of the topography dataset may affect overtopping calculations in two ways: 1) the limitation in the intertidal area (foreshore) which has an influence on the computation of coastal slope, and 2) the resolution of coastal features (maximum coastal elevations) in addition to issues in the absolute elevation (floating DEM relative to sea level). The capabilities of AW3D30 to represent coastal topography has been investigated in detail by Diaz et al.[1] at Capbreton, SW France and compared to other satellite-derived topography data sets. Diaz et al.'s[1] results show that AW3D30 has good skills to reproduce the topography of the coastal zone, with an overall good estimate of absolute elevation (a major improvement compared to previous products such as MERIT) because of its correction with ICESat-1. AW3D30 was found to be particularly capable of estimating coastal elevation maxima such as dunes. The foreshore area is generally lacking from the dataset, which is an artefact of the optical methodology of stereoscopy employed by AW3D30 on the ALOS mission, the low texture found in the foreshore zone, and rather uniform optical characteristics which preclude the method from finding homolog points in pairs of images. The global validation of AW3D30 is beyond the scope of this manuscript. An assessment of the uncertainty associated to the product is however necessary to gain confidence in our overtopping calculations. To this end, here the results obtained using AW3D30 are compared with two different independent datasets. The MERIT topo-bathymetry dataset[2] is here used to obtain two different estimates of coastal slopes to feed into wave runup calculations: (i) the coastal slope from the shoreline to the maximum subaerial coastal elevation, indicated as MERIT (topography) in **Figure S2**, and (ii) the foreshore slopes computed from the depth-of-closure to the shore (see Athanasiou et al.[2]), indicated as MERIT (foreshore) in **Figure S2**. Runup was then ultimately computed using 3 different coastal slopes: AW3D30; MERIT (topography): and MERIT (foreshore). The global distribution of these 3 different coastal slopes is shown in **Figure S2,** showing that coastal slopes derived from MERIT (foreshore) are milder compared to that derived from AW3D30 and MERIT (topography).

In terms of maximum coastal elevation, in addition to AW3D30 and MERIT, the FLOod PROtection Standards FLOPROS[3] dataset was used to obtain a third estimate of maximum coastal elevations and overtopping[4]. FLOPROS is generated by using flood exceedance levels from national protection policies, excluding overtopping. This is different from satellite radar-based and optical-based MERIT and AW3D30 products, with the result that FLOPROS derived coastal protection levels are significantly lower than MERIT (topography) and AW3D30 as shown in **Figure S3**.

The sensitivity of the globally aggregated annual number of overtopping hours ($N_{a,g}$) projections to the topographic dataset used is investigated here for different global mean sea level rise values and for different combinations of coastal slopes and maximum coastal elevations. **Figure S4** indicates that both FLOPROS and MERIT (topography) data bases generally provide lower estimates of maximum subaerial coastal elevation compared to AW3D30, which leads to more overtopping when FLOPROS or MERIT is used for elevations in the overtopping computations (**Figure S4**). Interestingly, when MERIT (topography) and MERIT (foreshore) are used for h maximum coastal elevation and coastal slope respectively, projected overtopping appears to be very close to that obtained when using AW3D30 for both parameters (**Figure S4**). The $N_{a,g}$ projections obtained when using AW3D30 for both coastal slopes and maximum coastal elevations is roughly in the middle of the range of projections obtained with different parameter/data set combinations, providing confidence to undertake our analysis with AW3D30 derived parameters. This global sensitivity analysis, which is more than any validation that is currently possible due to the scarcity of ground truth, provides a cross-evaluation among different topographical dataset,

and a range of uncertainty, indicating that the analysis undertaken here, exclusively using the AW3D30 data set for all topography related inputs to the overtopping computations is robust at the global scale of this study.

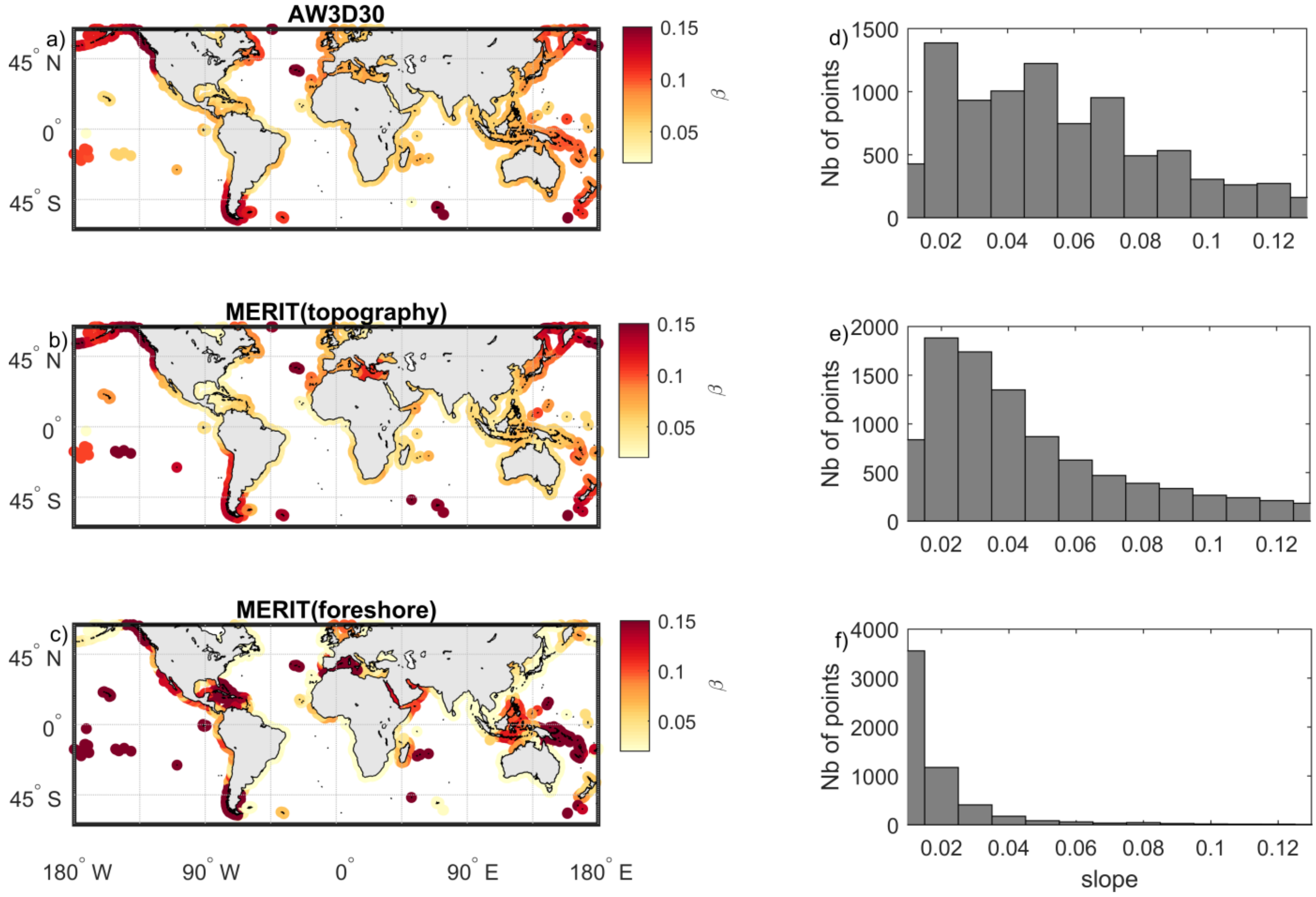


**Figure S2.** Global distribution of coastal slopes computed from 3 different datasets: AW3D30 (ALOS), MERIT (topography) and MERIT (foreshore). Left panels show the global spatial distributions. Right panels show occurrence histograms for different coastal slopes. Subaerial maxima of AW3D30 and MERIT are computed as the maximum elevation found from the first local maxima, and slopes are calculated from the shoreline to the maximum coastal elevation point (also see Data and Methods).

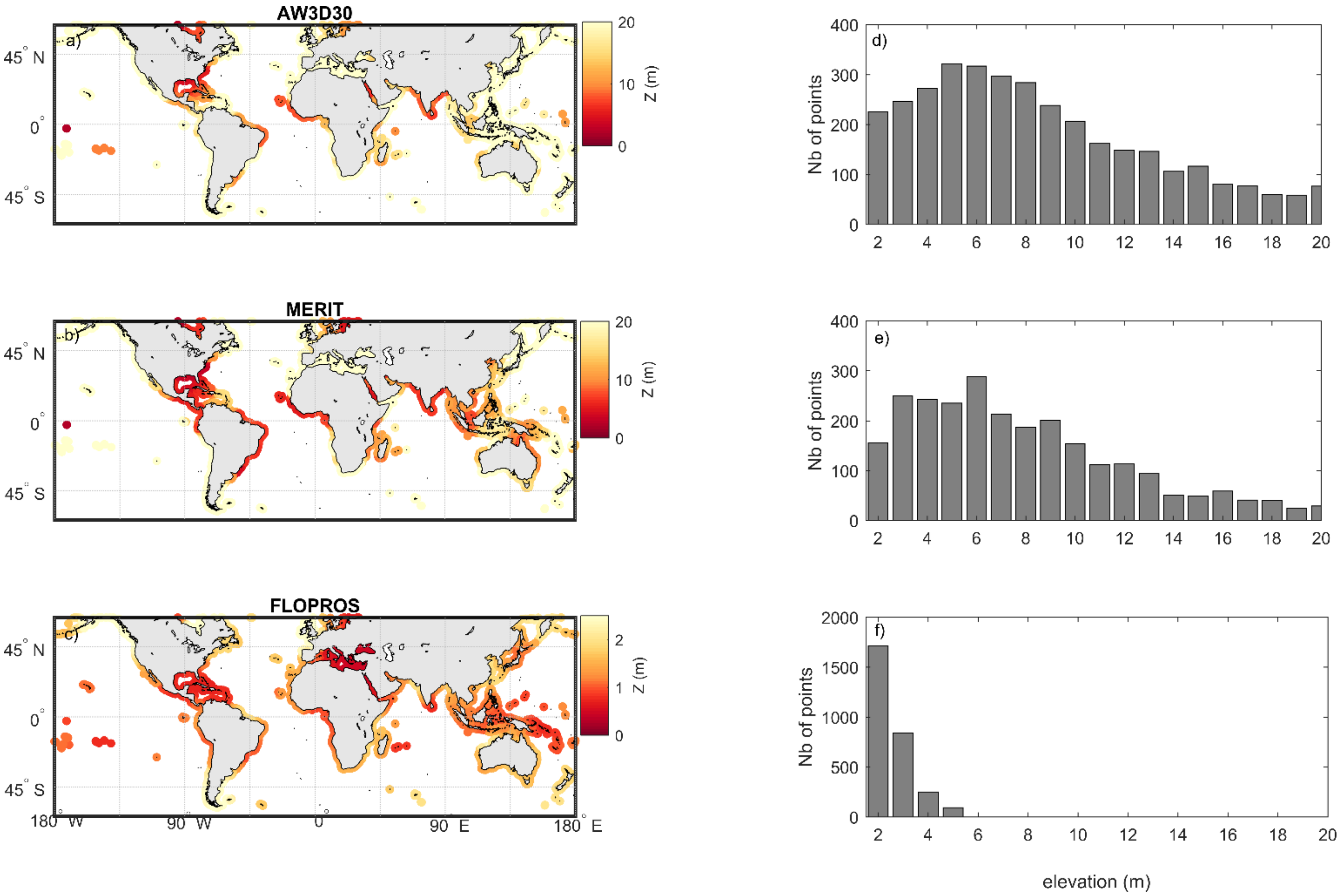


**Figure S3.** Global distribution of the maximum subaerial coastal elevation computed from 3 different datasets: AW3D30, MERIT, and FLOPROS. Left subpanels show global spatial distributions, Right column subpanels show occurrence histograms for different elevations.

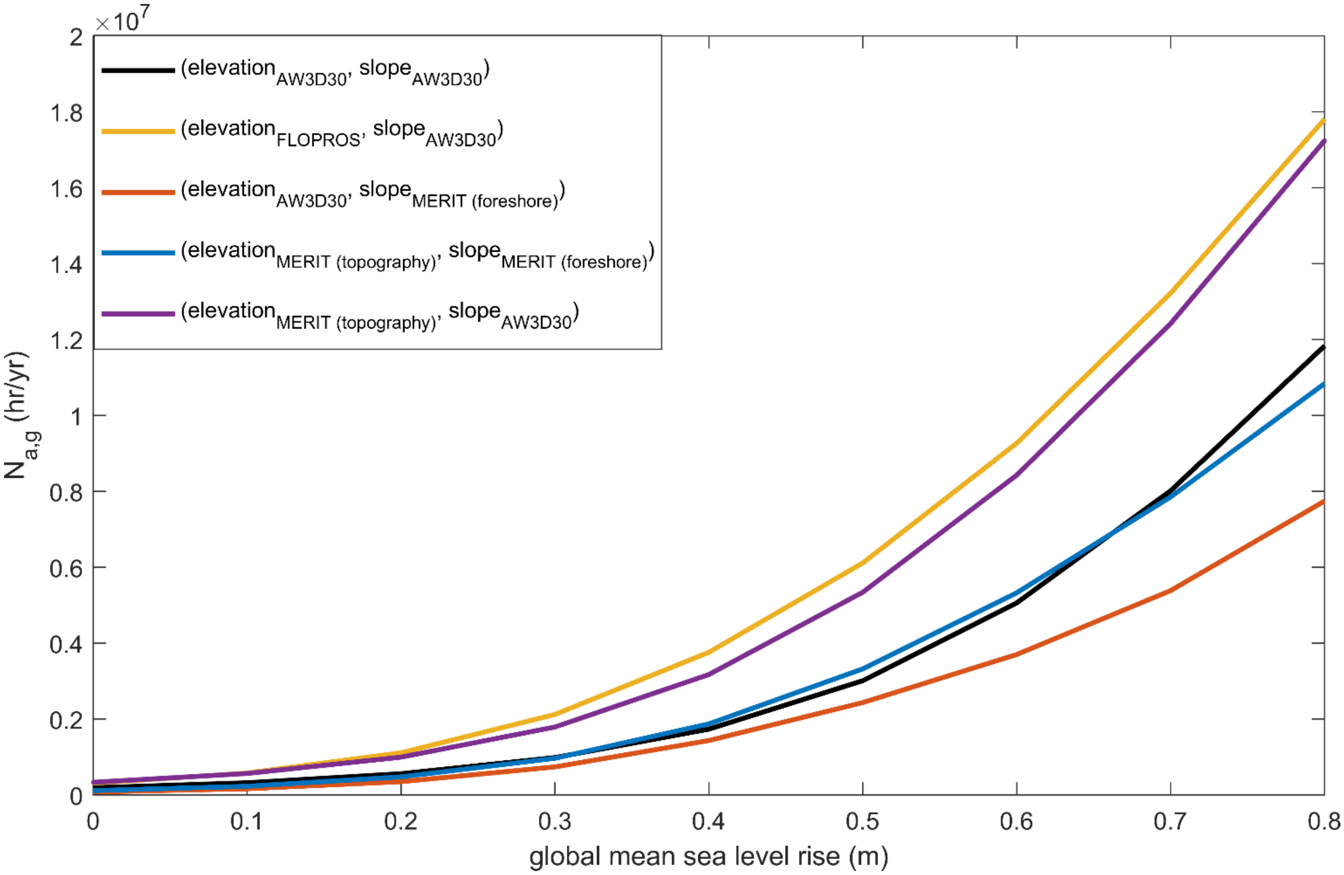


**Figure S4.** Sensitivity of the globally aggregated annual number of overtopping hours ($N_{a,g}$) to different coastal topography datasets. The dataset ultimately used in this study for all topographic parameters is AW3D30 (black line in figure).

**S3. Validation of the overtopping computations for historical coastal flooding events**

Here, the methodology adopted to compute overtopping at the global scale is tested for four documented major coastal flooding events (**Figure S5**) along the Atlantic coast of Europe (**Figure S5.a,** Xynthia storm in France[5]), Gulf of Mexico (**Figure S5.b**, Hurricane Katrina hurricane in USA[6]), Mediterranean South-East coast (**Figure S5.c,** Nile delta in Egypt[7–9]), Gulf of Guinea, West Africa (**Figure S5.d,** Lagos in Nigeria[10,11]) and Majuro in Pacific Marshall Islands (**Figure S5.e**[12]). The primary goal is to assess whether our method is able to reproduce the extreme still water level (ESWL) by comparing our calculations with sea level tide-gauge time series from the Global Extreme Sea Level Analysis (GESLA) dataset[13], which has already been validated globally by Melet et al.[14] (Figure S4 therein). The Extreme coastal water level is then computed by adding wave runup (from using local coastal slope and offshore waves in Stockdon et al.'s[15] parametrization) to the ESWL.

The second objective here is to determine the capabilities of the methodology to capture these observed historical overtopping/flooding major events from the comparison of computed extreme coastal water levels with these sea levels. Overtopping thresholds are computed from regional topography maxima and are aimed at describing regional characteristics, not local features. As stated in the main manuscript, we do not intend here to describe small scale topography such as, for e.g. dune breaching (for instance during Xynthia) and overflow due to local depressions in coastal elevation maxima (such as harbors, inlet), local shoreface singularities (e.g. coral reefs, convergence and sheltering, refraction/diffraction), but to detect regional characteristics of coastal topography and the general exposure to potential

overtopping potential. Process-based numerical simulations perform better in capturing small-scale local behavior (in particular wave processes such as infragravity energy transfer – see Bertin et al.[16]. Here our focus is on regional and temporal patterns which fall within the scope of this study. The computed ESWL (ECWL minus wave runup) estimate (dashed black line) show a good agreement with GESLA tidal gauge derived ESWLs (thin black). It should be noted that that ESWL alone cannot explain the overtopping and flooding observed in these case studies. It is only when wave runup is added that the water level exceeds the coastal elevation maxima for these 5 events. This is evident for the Gulf of Guinea case (**Figure S5.d**) where large tidal (spring) amplitude alone does not induce overtopping, but overtopping does occur even at lower tidal amplitude with concurrent large waves. This is particularly the case for Lagos and Pacific Islands events where the flooding is due to distant swell[12,17] in contrast to local storms associated with strong winds and surge (e.g. Xynthia storm in France and Katrina in US). This comparison with historical events together with the global sensitivity analysis of overtopping computations to different topography datasets and the regional validations in Figure M1 (in Data and Methods of the main article) provide sufficient confidence in the ability of the methodology implemented in this study to capture the salient characteristics of coastal overtopping at regional to global scale.

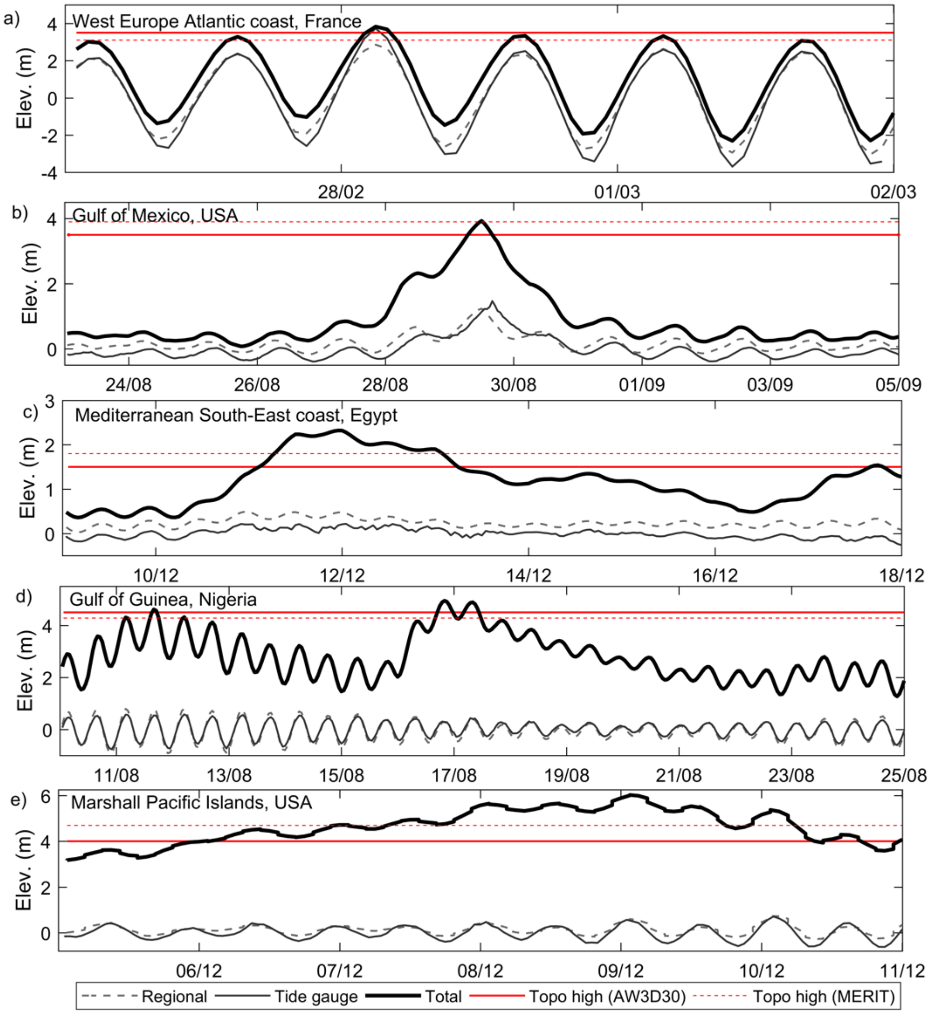


**Figure S5.** Validation of overtopping event detection with reported coastal flooding events: a) along the Atlantic coast of Europe (Xynthia storm in France[5]), b) Gulf of Mexico (Hurricane Katrina in USA[6]), c) Mediterranean South-East coast (Nile delta in Egypt[7–9]), d) Gulf of Guinea, West Africa (Lagos in Nigeria[10,11]) and e) Majuro in Marshall Pacific Islands, USA[12]. The computed Extreme Still Water Level ESWL (ECWL minus wave runup) estimate (dashed black line) compares well with that obtained from GESLA tidal gauges (thin black line). Comparison of the computed Extreme Coastal Water Level (ECWL), including wave runup (thick black line) with the regional maximum coastal elevation (red continuous lines) from AW3D30, shows the crucial influence of wave runup on overtopping. The MERIT derived maximum coastal elevation is also shown (red dash) for comparison.

REFERENCES


1. DIaz, H., Almar, R., Bergsma, E. W. J. & Leger, F. On the use of satellite-based Digital Elevation Models to determine coastal topography. *Int. Geosci. Remote Sens. Symp.* 8201–8204 (2019). doi:10.1109/IGARSS.2019.8899189
2. Athanasiou, P. *et al.* Global distribution of nearshore slopes with implications for coastal retreat. *Earth Syst. Sci. Data Discuss.* 1–29 (2019). doi:10.5194/essd-2019-71
3. Scussolini, P. *et al.* FLOPROS: an evolving global database of flood protection standards. *Nat. Hazards Earth Syst. Sci.* **16**, 1049–1061 (2016).
4. Vousdoukas, M. I. *et al.* Global probabilistic projections of extreme sea levels show intensification of coastal flood hazard. *Nat. Commun.* **9**, 2360 (2018).
5. Bertin, X., Bruneau, N., Breilh, J. F., Fortunato, A. B. & Karpytchev, M. Importance of wave age and resonance in storm surges: The case Xynthia, Bay of Biscay. *Ocean Model.* **42**, (2012).
6. Fritz, H. M. *et al.* Hurricane Katrina storm surge distribution and field observations on the Mississippi Barrier Islands. *Estuar. Coast. Shelf Sci.* **74**, (2007).
7. Frihy, O. E., Deabes, E. A. & El Gindy, A. A. Wave climate and nearshore processes on the mediterranean coast of Egypt. *J. Coast. Res.* **26**, (2010).
8. Refaat, M. M. & Eldeberky, Y. Assessment of Coastal Inundation due to Sea-Level Rise along the Mediterranean Coast of Egypt. *Mar. Geod.* **39**, (2016).
9. Ismail, N., Iskander, M. & El-Sayed, W. Assessment of coastal flooding at southern Mediterranean with global outlook for lowland coastal zones. *Coast. Eng. Proc.* **1**, (2012).
10. Nwilo, P. C. Managing the impacts of storm surges on Victoria Island, Lagos, Nigeria. *IAHS-AISH Publ.* (1997).
11. Olaniyan, E. & Afiesimama, E. . *Understanding ocean surges and possible signals over the Nigerian coast: a case study of the Victoria island bar-beach Lagos*. (2003).
12. Hoeke, R. K. *et al.* Widespread inundation of Pacific islands triggered by distant-source wind-waves. *Glob. Planet. Change* **108**, (2013).
13. Woodworth, P. L. *et al.* Towards a global higher-frequency sea level dataset. *Geosci. Data J.* **3**, 50–59 (2016).
14. Melet, A., Meyssignac, B., Almar, R. & Le Cozannet, G. Under-estimated wave contribution to coastal sea-level rise. *Nat. Clim. Chang.* **8**, 234–239 (2018).
15. Stockdon, H. F., Holman, R. A., Howd, P. A. & Sallenger, A. H. Empirical parameterization of setup, swash, and runup. *Coast. Eng.* **53**, 573–588 (2006).
16. Bertin, X. *et al.* Infragravity waves: From driving mechanisms to impacts. *Earth-Science Reviews* **177**, (2018).
17. Ford, M., Merrifield, M. A. & Becker, J. M. Inundation of a low-lying urban atoll island: Majuro, Marshall Islands. *Nat. Hazards* **91**, (2018).
